# Poly Organotin Acetates against DNA with Possible Implementation on Human Breast Cancer

**DOI:** 10.3390/ijms19072055

**Published:** 2018-07-14

**Authors:** George K. Latsis, Christina N. Banti, Nikolaos Kourkoumelis, Constantina Papatriantafyllopoulou, Nikos Panagiotou, Anastasios Tasiopoulos, Alexios Douvalis, Angelos G. Kalampounias, Thomas Bakas, Sotiris K. Hadjikakou

**Affiliations:** 1 Section of Inorganic and Analytical Chemistry, Department of Chemistry, University of Ioannina, 45110 Ioannina, Greece; glatsis@icloud.com; 2 Medical Physics Laboratory, Medical School, University of Ioannina, 45110 Ioannina, Greece; 3 Department of Chemistry, University of Cyprus, 1678 Nicosia, Cyprus; constantina.papatriantafyllopo@nuigalway.ie (C.P.); panagiotou.nikos@ucy.ac.cy (N.P.); atasio@ucy.ac.cy (A.T.); 4 Mössbauer Spectroscopy and Physics of Material Laboratory, Department of Physics, University of Ioannina, 45110 Ioannina, Greece; adouval@cc.uoi.gr (A.D.); tbakas@uoi.gr (T.B.); 5 Physical Chemistry Laboratory, Department of Chemistry, University of Ioannina, 45110 Ioannina, Greece; akalamp@cc.uoi.gr

**Keywords:** biological inorganic chemistry, acetic acid, organotins, bio-polymer, anti-cancer activity, cell cycle

## Abstract

Two known tin-based polymers of formula {[R_3_Sn(CH_3_COO)]*_n_*} where R = *n*-Bu– (**1**) and R = Ph– (**2**),were evaluated for their in vitro biological properties. The compounds were characterized via their physical properties and FT-IR, ^119^Sn Mössbauer, and ^1^H NMR spectroscopic data. The molecular structures were confirmed by single-crystal X-Ray diffraction crystallography. The geometry around the tin(IV) ion is trigonal bi-pyramidal. Variations in O–Sn–O···Sn′ torsion angles lead to zig-zag and helical supramolecular assemblies for **1** and **2**, respectively. The in vitro cell viability against human breast adenocarcinoma cancer cell lines: MCF-7 positive to estrogens receptors (ERs) and MDA-MB-231 negative to ERs upon their incubation with **1** and **2** was investigated. Their toxicity has been studied against normal human fetal lung fibroblast cells (MRC-5). Compounds **1** and **2** exhibit 134 and 223-fold respectively stronger antiproliferative activity against MDA-MB-231 than cisplatin. The type of the cell death caused by **1** or **2** was also determined using flow cytometry assay. The binding affinity of **1** and **2** towards the CT-DNA was suspected from the differentiation of the viscosity which occurred in the solution containing increasing amounts of **1** and **2**. Changes in fluorescent emission light of Ethidium bromide (EB) in the presence of DNA confirmed the intercalation mode of interactions into DNA of both complexes **1** and **2** which have been ascertained from viscosity measurements. The corresponding apparent binding constants (*K*_app_) of **1** and **2** towards CT-DNA calculated through fluorescence spectra are 4.9 × 10^4^ (**1**) and 7.3 × 10^4^ (**2**) M^−1^ respectively. Finally, the type of DNA binding interactions with **1** and **2** was confirmed by docking studies.

## 1. Introduction

Platinum-based compounds are at the focal point of research on potent anticancer drugs since the discovery of the anticancer potential of cisplatin, back during 70’s [1,2,3,4]. However, platinum-based cancer treatments are being dominated by serious side effects [5]. Moreover, cancer cells resistance against platinum based drugs is developed in a short while [6]. Nowadays, organometallic compounds, with a different pharmacological profile than that of platinum one, are developed and tested against various types of cancer cells [7].

During the last decades the anticancer activity of organotin compounds (OTCs) has been well studied [8,9,10,11,12,13,14,15,16,17,18,19,20,21,22,23,24,25,26,27,28,29]. Therefore, the investigation of new OTCs of low toxicity and improved anticancer activity are known to induce apoptosis in several cancer cell lines [17,18,19]. Their activity can be coupled to the lipophilicity of alkyl or aryl groups attached to the tin atoms [21]. In addition, due to their lipophilicity, OTCs are able to permeate membranes and reach the cell nucleus, where the dissociable ligands yield intermediate molecules capable of binding DNA [16,26].

The advantages of the use of polymeric drugs as anticancer agents have been described earlier [30]. This is because: (i) the polymers overcome cellular resistance mechanisms; (ii) the polymers could be used as carriers in high-dose chemotherapy; (iii) polymers are filtered out by the kidneys more slowly than small compounds increasing the body retention time; (iv) the size and structure of the polymer provide more binding sites to cellular targets; (v) the polymers can be act as hybrid drugs incorporating multiple anticancer agents against cells through different mechanisms; and (vi) the polymers accumulate in solid tumors more than in normal tissues [30].

In the course of our studies on the design and synthesis of new metallodrugs [13,14,15,16,17,18,19,20,21,22,23,24,25,26,27,28,29], the known {[R_3_Sn(CH_3_COO)]*_n_*} where R = *n*-Bu– (**1**) and R = Ph– (**2**) compounds were isolated from the reaction between acetic acid with tributyltin or triphenyltin oxides. The compounds were characterized via their physical properties and their FT-IR, ^119^Sn Mössbauer, and ^1^H NMR spectroscopic data, while their structures were verified by single-crystal X-Ray diffraction crystallography. The enhancement on the biological activity against tumor cells of the polymeric **1** and **2** is studied in relation to their polymeric intermolecular architecture (helical and zig-zag). The presence of acetic acid is also, expected to adjust the lipophilicity of the metallodrugs. The in vitro cell viability against MCF-7 (estrogen receptor (ER) positive) and MDA-MB-231 (estrogen receptor (ER) negative) was evaluated. Their genotoxicity has been studied against normal human fetal lung fibroblast cells (MRC-5). The type of the cell death caused by **1** and **2** was studied by flow cytometry assay. Finally, conclusions on Structure Activity Relationship are derived, in the light of the results obtained for OCT’s from our group up to now.

## 2. Results and Discussion

### 2.1. General Aspects

**Scheme 1:**
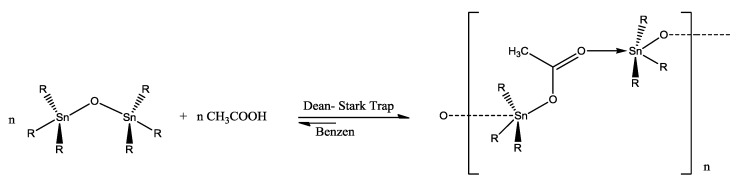
Preparation route of **1** and **2**.

Complexes **1** and **2** were synthesized as pale white powders by refluxing a benzene solution of tri-aryl-tin oxide and acetic acid (glacial) in a 1:1 molar ratio (Scheme 1), using a Dean–Stark water trap.

The formulae of **1** and **2** were first deduced by melting point and spectroscopic data. Crystals of the complexes **1** and **2** are stable in air. Complexes **1** and **2** are soluble in DMSO, DMF, toluene, ethanol, acetone, and diethyl-ether.

### 2.2. Solid State

#### 2.2.1. Vibrational Spectroscopy

The ν_as_(COO^−^) vibrations are observed at 1584 (**1**) and 1574 (**2**) cm^−1^ respectively, while the bands at 1418 (**1**) and 1416 (**2**) cm^−1^ are assigned to *ν*_s_(COO^−^) (Figure S1). The Δν [ν_as_(COO^−^) − ν_s_(COO^−^)] value is 166 (**1**) and 158 (**2**) cm^−1^, respectively. Monodentate coordination of the carboxylic group results in significantly higher difference values Δ*v* than those observed for the ionic compounds of the ligand [20], while when the ligand chelates, the Δ*v* is considerably smaller than that observed for its ionic compounds. For asymmetric bidentate coordination, the values are in the range of monodentate one [20]. When the –COO*−* group bridges metal ions, the Δ*v* values are higher than that of the chelating mode and nearly the same as that observed for ionic compounds [20]. In the case of sodium acetate, the Δ*v*[*v*_as_(COO*−*)−*v*_s_(COO*−*)] value is 170 cm*^−^*^1^ [31]. Since the Δ*ν* values in **1** and **2** (166 (**1**) and 158 (**2**) cm^−1^) is in the range of the corresponding one of sodium acetate (170 cm^−1^) the bridging coordination mode is concluded for the carboxylic group in **1** and **2** (Figure S1). Bands at 493 (**1**) and 457 (**2**) cm^−1^ in the spectra of **1** and **2** are assigned to the ν(Sn–O) bond vibrations [20], while the corresponding bands at 672, 614 (**1**) and 730, 698 (**2**) cm*^−^*^1^ are assigned to the antisymmetric and symmetric vibrations of Sn–C bonds [29].

#### 2.2.2. ^119^Sn Mössbauer Spectroscopy

**Figure 1:**
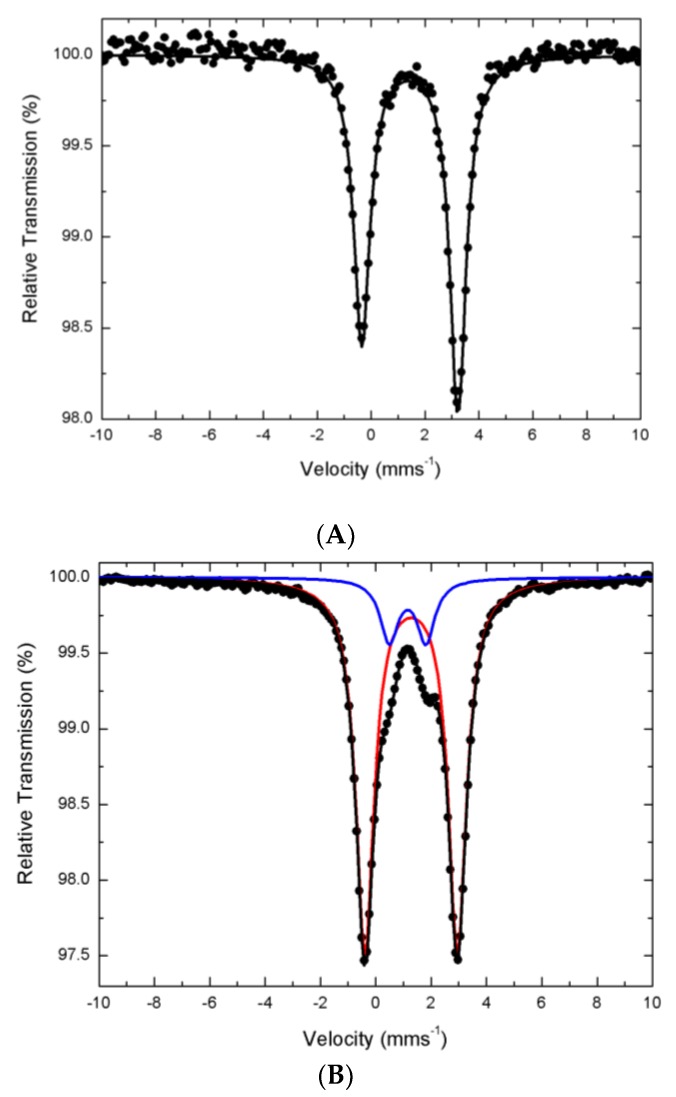
^119^Sn Mössbauer spectra of **1** (**A**) and **2** (**B**) at 80 K.

^119^Sn Mössbauer spectra at 80 K are shown in Figure 1.

The spectrum of **1** consists of one asymmetric Lorentzian doublet. The absorption line intensity asymmetry could be attributed to the recoilless fraction (f) asymmetry, which could be a consequence of vibrational-bond anisotropic involving the Sn ions [32]. Preferred orientation of the crystallites in the powdered sample cannot also be excluded in order to justify this asymmetry. The occurrence of one Lorentzian double, indicates either the existence of one type of Sn atom in **1** or one structural isomer [25,29]. The corresponding spectrum of **2** consists by two symmetric Lorentzian doublets. The occurrence of two symmetric Lorentzians, however, indicates two kinds of tin centers under different environment in **2** with 85–15% molar ratio [25,29]. The values of the Isomer Shifts (I.S.) of +1.44 (**1**), 1.28 (**2A**) and 1.15 (**2B**) mm·s^−1^ corresponds to the (4+) oxidation state [25,29]. The quadrupole splitting parameter (ΔΕq) values are 3.57 (**1**), 3.35 (**2A**) and 1.33 (**2B**) mm·s^−1^. Therefore, trigonal-bibyramidal (tbp) geometry should be concluded for tin(IV) ions in **1** and **2A** as in the case of tbp R_3_Sn(IV) (eg-R_3_ = alkyl) geometry where the ΔΕq values lie between 2.50–4.00 mm·s^−1^ [25,29]. On the contrary, tetrahedral (tet) geometry R_3_Sn(IV) results in to ΔΕq values of 1.30–3.00 mm·s^−1^ [25,29], tet conformation should be attributed to the tin(IV) atoms in **2B**.

#### 2.2.3. Crystal and Molecular Structures of [Bu_3_SnCH_3_COO]*_n_* (**1**) and [Ph_3_SnCH_3_COO]*_n_* (**2**)

**Figure 2:**
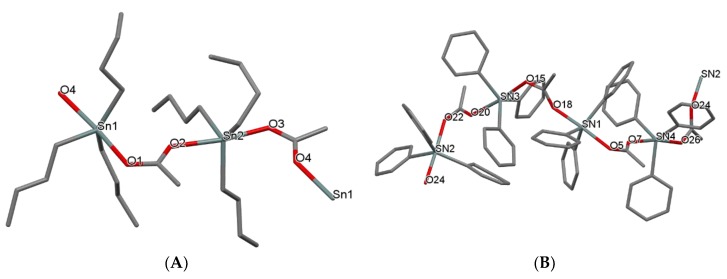
Molecular diagrams of **1** (**A**) and **2** (**B**).

Crystals suitable for X-ray analysis were obtained by slow evaporation of diethyl-ether solutions of **1** and **2**. Their formula was confirmed here by single crystal X-ray diffraction analysis at ambient conditions. The structure of **1** is identical to that already reported by M. Adeel Saeed et. al. [33]. Thus **1** crystallizes in P2_1_/c space group, *a* = 10.1845(3), *b* = 20.2542(7), *c* = 16.2466(6) Å, β = 94.739(3)°, *V* = 3339.87 Å^3^; while the reported one crystallizes in P2_1_/c space group, *a* = 10.386(4), *b* = 20.924(3), *c* = 16.584(6) Å, β = 92.87(2)°, *V* = 3599(2) Å^3^ [33]. Although, **2** crystallizes in Pn space group with *a* = 16.7427(6), *b* = 10.0426(2), *c* = 25.5119(8) Å, α = 89.999(2), β = 100.936(3), γ = 89.998(2)°, *V* = 4211.68 Å^3^, the already reported crystallizes in P2_1_/c, space group, *a* = 8.969(4), *b* = 10.146(5), *c* = 19.540(7) Å, β = 93.70(4)°, *V* = 1774.5 Å^3^ [34] suggesting a polymorphism between **2** and the published one. However, the extended disorder on the density observed in **2** prevent its accurate refinement allowing only qualitative conclusions to be drawn. Although the obtained X-ray data do not allow discussion about bond lengths and angles, they are of sufficient quality to determine the connectivity and the packing of **2**. The molecular diagrams of **1** and **2** are shown in Figure 2.

**Scheme 2:**
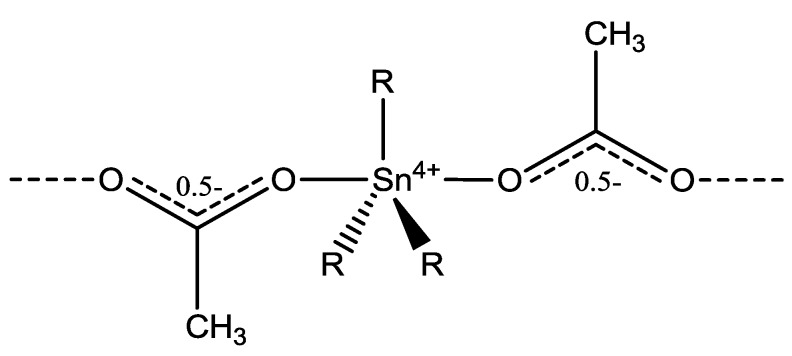
Charge distribution.

Three C atoms from the alkyl groups and two O atoms from two de-protonated CH_3_COOH molecules form the trigonal bipyramidal arrangement around the Sn ions in **1**. The average C–O bond lengths found in **1** and **2** (1.260 ± 0.020 Å), indicates a bond order of 1.5 e. This delocalization of the electron density in the –COO^−^ group suggests a charge distribution shown in Scheme 2.

### 2.3. Solution Studies

#### ^1^H-NMR Spectrοscopy

The ^1^H-NMR spectrum of free acetic acid in DMSO-*d_6_* is dominated by a single resonance signal at 1.91 (s, H) ppm for the methyl protons, which is shifted upon its coordination to the tin(IV) ion at 1.776 (s, H) ppm in **1** and at 1.758 (s, H) ppm in **2** (Figure S2). In the case of **1** four additional signals are observed at 1.52 ppm (t, H^a^(–^a^CH_2_–Sn)), 1.27 ppm (m, H^b^(–^b^CH_2_–CH_2_–Sn)), at 1.00 ppm (m, H^c^((–^c^CH_2_–CH_2_–CH_2_–Sn)), and 0.84 ppm (t, H^d^ ((^d^CH_3_–CH_2_–CH_2_–CH_2_–Sn)) of the butyl substituent bind on tin(IV) ions (Figure S2). These four signals (1.52–0.84 ppm) have been replaced by the signals at 7.79–7.45 ppm in the spectrum of **2** (Figure S2), which were attributed into the aromatic protons of phenyl substituent of organotin moieties. Since the cells were incubated for 48 h the stability of **1** and **2** is checked for this period with ^1^H-NMR spectroscopy. No changes were observed between the initial spectra of freshly prepared solutions and the corresponding spectra when measured after 48 h confirming the retention of the structures in solution (Figure S2).

### 2.4. Biological Tests

#### 2.4.1. Anti-Proliferative Activity

Organotin compounds **1** and **2** were tested for their in vitro cytotoxic activity against human breast adenocarcinoma cell lines, MCF-7 and MDA-MB-231, by the mean of sulforhodamine B (SRB) assay [16,17]. The cells were incubated for 48 h with **1** and **2**. Since ERs are expressed in 65% of human breast cancer, (a hormone dependent malignancy) the MCF-7 and MDA-MB-231 cells were used in order to ascertain the influence of the ER’s in the mechanism of action of **1** and **2** [16,17]. MCF-7 cells serve as a valuable model system to elucidate pathways of hormone response and resistance. Especially, the MCF-7 cells were used for studying estrogen response both in vitro and in vivo [35]. MDA-MB-231 human breast cancer cells, on the other hand, are used as a model of ER-negative breast cancers [36].

The IC_50_ values of **1** and **2** against MCF-7 cells lie in the range of nM and they are 0.25 ± 0.02 and 0.21 ± 0.01 μΜ respectively, while their corresponding IC_50_ values against MDA-MB-231 cells are 0.20 ± 0.01 and 0.12 ± 0.01 μΜ. The activity of **1** follows reverse order to the corresponding one of **2** against these cell lines suggesting no interference of the estrogen receptors to their mechanism. By taking into account the IC_50_ value of cisplatin against MCF-7 and MDA-MB-231 cells (5.5 ± 0.4 and 26.7 ± 1.1 μΜ respectively), both **1** and **2** exhibit extremely cytotoxic activity against these cell lines. These values indicate 22 and 26-fold higher activity of **1** and **2** against MCF-7 cells than cisplatin and 134 and 223-fold against MDA-MB-231 cells. Despite their strong activity against tumor cells, **1** and **2** also exhibit high toxic activity against MRC-5 cells with IC_50_ values of 0.22 ± 0.01 (**1**) and 0.11 ± 0.01 (**2**) μΜ, respectively. The IC_50_ values against MCF-7, MDA-MB-231, and MRC-5 cells of organotin compounds studied from our group, are summarized in Table 1. The following conclusions are made: (i) tri-organotin derivatives are more active than the corresponding di-organtin ones; (ii) organotin derivatives of carboxylic acids are generally more active than those of other types of ligands; (iii) organotin compounds are also highly toxic, even more toxic than cispaltin; (iv) however the selectivity index, which is defined as the IC_50_ value against MRC-5 towards the corresponding value against MCF-7, and is an indicator of the therapeutic potency of a compound (the higher the value is, the better potency), shows that **1** and **2** are more potent therapeutics (TPI values of 0.88 (**1**) and 0.52 (**2**)) than cisplatin (TPI = 0.20). (v) The tri-*n*-butyl tin compound of thiobarbituric acid (*n*-Bu)_3_Sn(*o*-HTBA)(H_2_O) exhibits the higher TPI value of 1.6 (Table 1).

#### 2.4.2. Evaluation of Genotoxicity by Micronucleus Assay In Vitro

Micronucleus assay is a reliable and an accessible technique to evaluate the appearance of genetic damage on a cell. The detection of micronucleus (MN) indicates mutagenic, genotoxic, or teratogenic effects [37]. In the presence of exogenous genotoxic factors, the MN is formed due to the metaphase–anaphase transition of the mitotic cycle. The possible induction of micronucleus frequencies was evaluated when MRC-5 cells were treated by **1** and **2** at the concentrations of their IC_50_ values. The micronucleus frequency in the MRC-5 cell culture without treatment is 0.91 ± 0.02%, while it is 1.0 ± 0.1% upon treatment with DMSO. However, the micronucleus frequencies are slightly increased when the cells are incubated with **1** and **2** to 2.10 ± 0.04% (**1**) and 2.19 ± 0.03% (**2**) (Figure S3). The compounds **1** and **2** show a slightly increase in micronucleus frequency in contrast to the control or to cisplatin (1.6%) at its IC_50_ value of 26 μΜ [37]. Cisplatin is used as a reference control. Doxorubicin has also been used as reference control from other groups. MRC-5 cells show slight increasing MN’s when they are treated with **1** and **2** than the case of cisplatin indicating higher genotoxocity indeed. However, the treatment of MRC-5 cells with 0.18 μΜ or 0.014 μΜ of doxorubicin increases the percent of micronucleus at (94.7 ± 20.0)% and (17.00 ± 1.73)%, respectively, in contrast to the control ones (15.0 ± 2.64)% [38]. Thus, despite its higher genotoxicity of **1** and **2** towards MRC-5 cells than cisplatin both exhibit significant lower MN percentage than doxorubicin a medication against tumors in humans in used. It is therefore considered that **1** and **2** are nongenotoxic substances.

#### 2.4.3. Cell Cycle Studies

Internucleosomal DNA fragmentation has been described as one of the main characteristics of the apoptotic process and can be identified by a sub-G_1_ peak on DNA frequency histograms [37]. Therefore, the apoptotic type of the cell deaths caused by the regulation of cell cycle progression because of **1** and **2** can be evaluated by flow cytometric analysis since these cells give a sub-G_1_ peak [37]. The percentage of cells in the phases of the cell cycle was analyzed after 48 h exposure of MCF-7 cells with **1** and **2** at their IC_50_ values.

**Figure 3:**
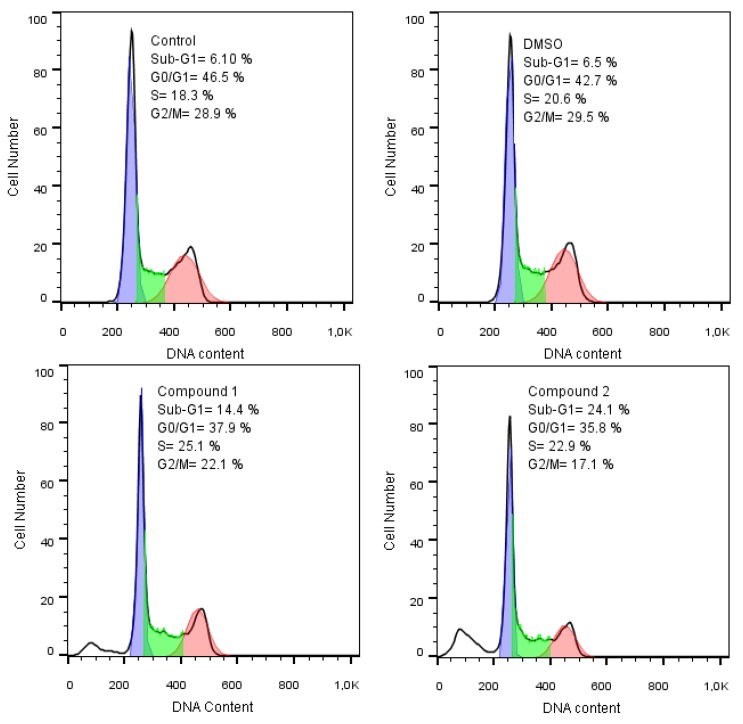
Number of MCF-7 cells in sub-G_1_, G_0_/G_1_, S, and G_2_/M phases, upon their treatment with **1** and **2**. The meaning of color labeling is white= Sub-G1, blue= G0/G1, green= S, pink= G2/M.

The effect on the cell cycle which is illustrated in Figure 3, as the number of cells towards DNA content in sub-G_1_, G_0_/G_1_, S, and G_2_/M phases is caused by **1** and **2**. The untreated cells are spread in 6.1% sub-G1 phase, 46.5% in G_0_/G_1_, 18.3% in S, and 28.9% in G_2_/M phases. After incubation of MCF-7 cells with **1** and **2**, a significant increase in the number of apoptotic cells in sub-G_1_ phase (14.4% (**1**), 24.1% (**2**), respectively) was observed towards the control group (6.1%). In the case of **1**, the cells in G_0_/G_1_ phase are reduced to 37.9%, on the contrary, with 46.5% for the untreated cells while in the case of **2**, the corresponding value decrease to 35.8%. However, the percentage of MCF-7 cells in S phase, was increased to 25.1% (**1**), 22.9% (**2**). Finally, the percentage of MCF-7 cells in G_2_/M phase was reduced to 22.1% (**1**) and 17.1% (**2**), respectively. In the DMSO-treated cells, the distribution in phases sub-G_1_ (6.5%), G_0_/G_1_ (42.7%), S (20.6%), and G_2_/M (29.5%) were similar to the corresponding ones of control cells’. All the data obtained in cell cycle studies of **1** and **2** are summarized in Table 2.

In conclusion, **1** and **2** stimulate S-phase cell cycle arrest, thus suppressing cell proliferation by inhibiting DNA synthesis, in accordance to other anticancer agents (resveratrol, mitomycin C, and hydroxyurea) [37]. Likewise, cisplatin causes cell cycle arrest at S and G_2_/M phases and the percentage of MCF-7 cells in sub-G_1_ phase is increased, exhibiting an increasing number of apoptotic cells [37]. To summarize, metal drugs of this type induce cell cycle arrest either in G_0_/G_1_ or in S phase (like cisplatin), resulting in MCF-7 cell growth inhibition. Therefore, the reduced cell growth caused by **1** and **2** is attributed to the apoptotic type of cell death, in accordance to the way of action of cisplatin [37].

#### 2.4.4. Detection of the Loss of the Mitochondrial Membrane Permeabilization (MMP Assay)

The releasing of cytochrome c in the cytosol through the loss of mitochondrial membrane permeability activates the mitochondrion cell apoptosis pathway [37]. The induction of loss in mitochondrial membrane permeability in tumor cells is one of the main accomplishments of targeted chemotherapy. The MMP assay is based on the cationic hydrophobic mitochondrial potential dye which accumulates in normal mitochondria. When cells are treated with a metallo-agent, the mitochondrial membrane permeability collapses, and the fluorescence emission of the dye decreases simultaneously.

The MCF-7 cells were treated with **1** and **2** at their IC_50_ values, for 48 h, and the fluorescence of the MMP assay dye decreased by 6.71% (**1**) and 6.52% (**2**), respectively. When the MCF-7 cells are treated with cisplatin at its IC_50_ values (5.5 μΜ) the fluorescence of the MMP assay dye decreased by 54.9% [37]. Therefore, the MMP assay should not support mitochondrial membrane permeability loss. Thus, **1** and **2** cause cell death by a different mechanism. Since apoptosis has been observed from cell cycle studies, it should be activated by a different mechanism than mitochondrion pathways.

#### 2.4.5. DNA Binding Studies

DNA is the main target of the successful chemotherapeutics, the interaction of **1** and **2** towards CT-DNA was investigated by viscosity measurements and fluorescence spectroscopic studies.

**Figure 4:**
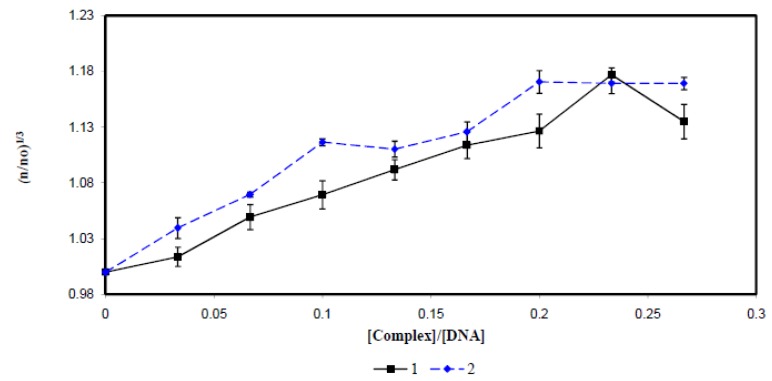
Effect of increasing concentrations of **1** and **2** on the relative viscosity of CT-DNA at 25 °C. ([DNA] = 10 mM, r = [compound]/[DNA], *n* is the viscosity of DNA in the presence of **1** or **2** and *n_o_* is the viscosity of DNA alone).

**Figure 5:**
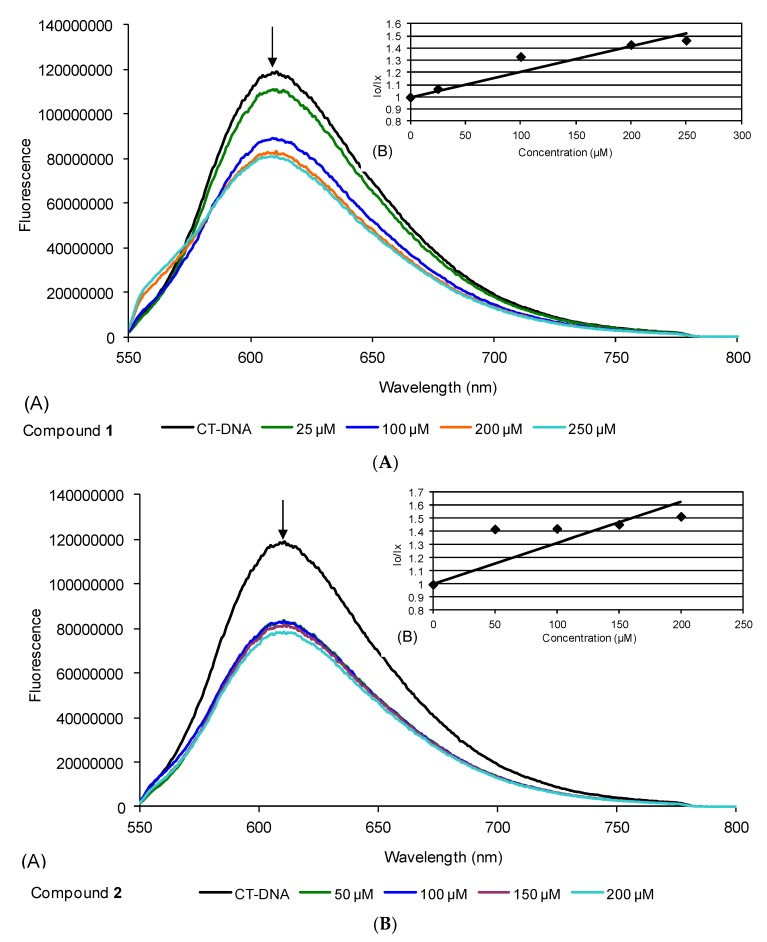
Emission spectrum of EB bound to DNA (peak around 610 nm) decreases in order of the concentration of the complex (**1** (**A**) and **2** (**B**)). The arrows show the intensity changing upon increasing complex concentration. Inset shows the plots of emission intensity Io/I vs. [complex].

**Figure 6:**
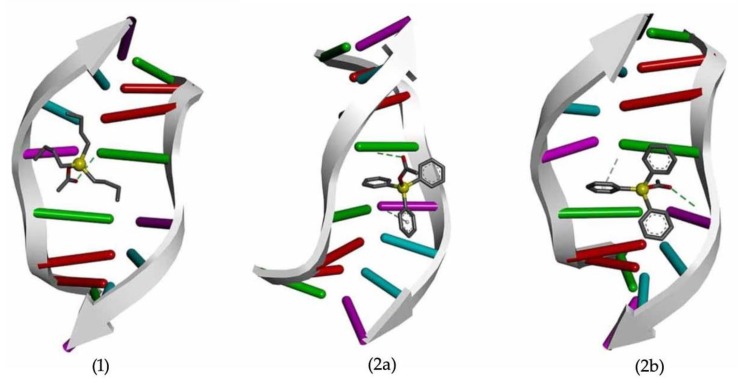
DNA docking and H-bonding interactions between B-DNA and compounds **1** and (**2a, 2b**).

(a) Viscosity measurements: DNA length changes upon its incubation with anticancer agents affect strongly affecting the viscosity of its solution. Thus (i) if the agent intercalates in the DNA strands, this results in its lengthening and a viscosity increase; (ii) if the agent interacts electrostatically with the DNA, no effect on DNA length is caused and therefore no significant change in viscosity is observed; (iii) however, in case the DNA strands are cleaved by an agent, the length of the DNA decreases, and also, the viscosity decreases significantly (iv) bending of the DNA helix caused by the agent reduces the viscosity. Therefore, viscosity exhibits high sensitivity to changes in the DNA and it is used for the study of the binding modes of an agent towards DNA [39]. The solution of CT-DNA (10 mM) is incubated with increasing amounts of **1** and **2** so that the [compound]/[DNA] molar ratio reaches *r* = 0.27. The relative viscosity of the solution which contains the agent/DNA/buffer, towards the corresponding one which contains DNA/buffer, increased for both compounds (Figure 4), suggesting an intercalation mode of interaction between **1** and **2** and CT-DNA.

(b) Fluorescence Spectroscopic Studies: In order to verify the intercalation mode of **1** and **2** towards CT-DNA, fluorescence spectroscopic studies were carried out. In the fluorescence spectroscopic studies the dye ethidium bromide (EB) is used. In the presence of DNA, EB emits, due to its strong intercalation between the adjacent DNA base pairs [37]. The displacement of EB during titration with the agent suggests an intercalative binding mode. The emission data of the solutions of EB with CT-DNA at 610 nm, with increasing concentrations of **1** and **2** (0–250 μΜ) upon their excitation at 532 nm, were recorded (Figure 5). The decreasing percent in fluorescence upon increasing of concentration of **1** and **2** at 610 nm was 31.5% (**1**) and 30.1% (**2**), respectively, confirming that both compounds can interact with DNA by the intercalation mode. The corresponding apparent binding constants (K*_app_*) of **1** and **2** towards CT-DNA calculated through fluorescence spectra are (4.9 ± 0.5) × 10^4^ (**1**) and (7.3 ± 1.3) × 10^4^ (**2**) M^−1^, respectively, indicating stronger binding affinity of the triphenyltin than the tri-*n*-butyltin acetates.

(c) Computational studies—molecular docking: In order to verify the type of interaction between DNA with **1** or **2** molecular docking calculations were performed. Small aromatic molecules can typically bind to DNA by intercalation. Generally, either a planar molecule or a fragment is inserted between two adjacent DNA base pairs to form a hydrophobic pocket in the DNA structure. This non-covalent interaction is usually stabilized by π-interactions; at the same time, additional interactions with the groves are possible. Statistically, the CG intercalation site is preferable for intercalation [40]. Upon ligand binding, the DNA structure is slightly elongated and accommodates a small gap between two consecutive base pairs for the intercalators. Consequently, the latter can act as potent antitumor drugs and mutagens, inhibiting DNA replication and transcription. Targeting DNA sequences is a challenging task for docking software, especially when intercalation sites are unavailable, because gap openings cannot be simulated with rigid receptors. Moreover, it has been shown that popular docking software fails to predict the intercalation into canonical B-DNA when analogous solved structures are lacking [41]. For these reasons, the DNA target chosen for our study was 1DSC (PDB: www.rcsb.org) which is an octamer d(GAAGCTTC)_2_ complexed with actinomycin D [42]. Actinomycin D is a potent antibiotic with high antibacterial and antitumor activity. It intercalates to DNA by localizing a phenoxazone ring at a GpC sequence while the two cyclic polypeptides of the drug bind to the DNA minor groove. 1DSC has been already used successfully for docking of metal complexes [43] and organic compounds [44] acting as DNA intercalators. The geometry of compounds **1** and **2** is trigonal bipyramidal (TBP) in the solid state. However, upon solvation in aqueous media their structure are converted to tetrahedral by cleavage of the Sn-O_(acetate)_ dative bond. Tetrahedral metal complexes, unlike those with TBP geometry, can effectively bind to DNA by intercalation, although they normally cannot penetrate as deep as square planar complexes [45]. The optimized structures of the two organotin compounds [R_3_Sn(CH_3_COO)], (R = *n*-Bu (**1**), Ph– (**2**)) were used as ligands for DNA docking using AutodockVina. Molecular docking evaluates affinity potentials through a precalculated grid finding favorable binding positions for a flexible ligand towards a rigid macromolecular target. Complexes **1** and **2** were successfully docked into the B-DNA duplex and the lowest energy poses are depicted in Figure 6. The tri-phenyl derivative can adopt two different conformations intercalating in the GC region through either one pnenyl-ring (Figure 6(**2a**)) or the carboxylic moiety (Figure 6(**2b**)) with computed binding free energies −5.5 and −5.1 kcal/mol, respectively. The planarity of the phenyl ring favors π-π stacking interactions with the DNA base pairs, while additional hydrogen bonding interactions with the carboxylate O (G4:N2-Lig O, 2.97 Å and Lig O-C5O4, 3.1 Å) and with the pi-orbitals of the phenyl ring (G12:N2-Lig pi, 2.96 Å and G4:N2-Lig pi, 3.6 Å). On the other hand, the minimum energy conformation of the tri-*n*-butyl-derivative shows a semi-intercalation into the same GC region (Figure 6(**1**)) with lower binding affinity (−3.7 kcal/mol). The structure is also stabilized by hydrogen bonding through the carboxylic moiety (G4:N2-Lig O, 3.13 Å). Possibly, the bulk and agile butyl groups significantly increase the steric hindrance and prevent the intercalation. These results support the experimental results showing that the phenyl derivative exhibits higher antitumor activity possibly through its intercalative mode of action (Table 1).

## 3. Experimental

### 3.1. Materials and Instruments

All solvents used were of reagent grade, (Aldrich, Merck, Darmstadt, Germany) and they were used with no further purification. Infrared spectra in the region of 4000–370 cm^−1^ were obtained in KBr pellets with a Jasco FT-IR-6200 spectrometer. The ^1^H-NMR spectra were recorded on a Bruker AC 250, 400 MHFT-NMR instrument in DMSO-*d_6_*. Chemical shifts are given in ppm using ^1^H-TMS as internal reference. Elemental analysis for C, H, N, and S were carried out with a Carlo Erba EA MODEL 1108 (Waltham, MA, USA). The ^119^Sn Mőssbauer spectra were collected at sample temperature of 80 K using a constant acceleration spectrometer equipped with a Ca^119m^SnO_3_ source kept at room temperature. The isomer shift values of the components used to fit the spectra are given relative to SnO_2_ at room temperature. The ^119^Sn Mőssbauer spectra were recorded with Constant acceleration WissEl-Wissenschaftliche Elektronik GmbH spectrometer (Starnberg, Germany).

### 3.2. Synthesis and Crystallization of {[(n-Bu)_3_Sn(CH_3_COO)]_n_} (**1**) and {[Ph_3_Sn(CH_3_COO)]_n_} (**2**)

Although the synthesis of these compounds is already known [33,34] we briefly described the procedure follows here. 0.5 mmol of tri*-n-*butyltin oxide (C_24_H_54_OSn_2_, 0.298 g) for **1**, or triphenyltin(IV) hydrooxide (C_24_H_16_OSn, 0.183 g) for **2**, were diluted with 0.5 mmol acetic acid, in 20 mL benzene in a 100-mL spherical flask. The flask was fitted with a Dean–Stark moisture trap and the reaction mixture was refluxed for 3 h. The solution was filtered and the clear filtrate was concentrated to dryness. Crystals of **1** and **2**, suitable for X-ray analysis, were formed by slow evaporation of a diethyl ether solution.

**1**: Yield: 40%; m.p: 75–76 °C; (C_14_H_30_O_2_Sn)*_n_*·(MW = 349.04); elemental analysis: found C = 48.23, H = 6.65%; calcd: C = 48.18, H = 8.66%. MID-IR (cm^−1^) (KBr): 3045 w, 2330 w, 2295 s, 1959 w, 1821 w, 1659 s, 1643 w, 1573 w, 1555 s, 1428 s, 1334 vs, 1261 vs, 1077 vs, 1025 w, 997 w, 729 w, 696 w, 665 w, 609 w, 499 w, 455 w. ^1^H NMR (ppm) in DMSO-*d_6_*: 1.776 (s), 1.578–1.501 (q), 1.327–1.236 (q), 1.043–1.001 (t), 0.880–0.843 (t).

**2**: Yield: 40%; m.p: 110–115 °C; (C_20_H_18_O_2_Sn)*_n_* (MW = 409.02); elemental analysis: found C = 58.91, H = 4.40%; calcd: C = 58.73, H = 4.43%. MID-IR (cm^−1^) (KBr): 2400 w, 1574 w, 1556 s, 1415 w, 1384 s, 1015 s, 867 w, 671 w, 612 s. ^1^H NMR (ppm) in DMSO-*d_6_*: 7.850–7.707 (q), 7.452–7.390 (q), 1.758 (single), 1.114–1.079 (t).

### 3.3. X-Ray Structure Determination

Single crystal X-ray diffraction data for **1** and **2** were collected on an Oxford-Diffraction Supernova diffractometer, equipped with a CCD area detector utilizing Cu *K*α (λ = (1.5418 Å)) radiation. A suitable crystal was mounted on a Hampton cryoloop with Paratone-N oil and transferred to a goniostat where it was cooled for data collection. Empirical absorption corrections (multiscan based on symmetry-related measurements) were applied using CrysAlis RED software [46]. The structures were solved by direct methods using SIR2004 [47] and refined on F^2^ using full-matrix least-squares with SHELXL-2014/7 [48]. Software packages used were as follows: CrysAlis CCD for data collection [46], CrysAlis RED for cell refinement and data reduction [46], WINGX for geometric calculations [49]. The non-H atoms were treated anisotropically, whereas the aromatic H atoms were placed in calculated, ideal positions and refined as riding on their respective carbon atoms. The single crystals of compound **2** exhibited a fairly poor diffraction pattern. As a result moderate quality X-ray data were collected which did not lead to a publishable crystal structure. For this reason the X-ray data of **2** are not quoted here and have not been deposited in Cambridge Structural Database.

Supplementary data (1_bu3sn2o_asp_checkcif_new.pdf and 1_bu3sn2o_asp_FINAL.cif) are available from CCDC, 12 Union Road, Cambridge CB2 1EZ, UK, (e-mail:deposit@ccdc.cam.ac.uk), on request, quoting the deposition numbers CCDC-1846946 (**1**).

**1**: (C_14_H_30_O_2_Sn)*_n_*: MW = 349.04, Monoclinic, space group P21/c, *a* = 10.1845(3), *b* = 20.2542(7), *c* = 16.2466(6) Å, β = 94.739(3)°, *V* = 3339.9(2) Å^3^, *Z* = 4, *T* = 100 K, ρ(calc) = 1.388 g·cm^−3^, μ = 1.522 mm^−1, *F*^(000) = 1440. 22782 reflections measured, 5864 unique (*R*_int_ = 0.041), 5131 with *I* > 2σ(*I*). The final *R*_1_ = 0.0265 (for 5131 reflections with *I* > 2σ(*I*)) and *wR_2_(F*^2^) = 0.0678 (all data), *S* = 1.09.

### 3.4. Biological Tests

Biological experiments were carried in dimethyl sulfoxide Dulbecco’s Modified Eagle’s Medium solutions (DMEM) DMSO/DMEM (0.02–0.2% *v*/*v*) for the complexes **1**–**2**. Stock solutions of the complexes **1**–**2**, (0.01 M) in DMSO were freshly prepared and diluted in with cell culture medium to the desired concentrations (0.05–0.4 μM). Results are expressed in terms of IC_50_ values, which is the concentration of drug required to inhibit cell growth by 50% compared to control, after 48 h incubation of the complexes towards cell lines. Since there is no “universal” correct time point to find the IC_50_ value of a given compound. Generally the inhibitory concentration of the 50% of the cells is determined at once at the time of the cells (of interest) doubling time, and twice the time of the doubling time. In cell culture, the vast majority of adherent cell lines display a doubling time between 18 and 24 h. In our research unit, we prefer to use 48 h since 24 h is too short to determine a reliable IC_50_ concentration. The cell viability was determined by SRB assay as previously described [37] and it is briefly described here: Cells were plated (100 μL per well) in 96-well flat-bottom microplates at various cell inoculation densities (MCF-7, MDA-MB-231 and MRC-5: 6000, 6000 and 2000 cells/well respectively). Cells were incubated for 24 h at 37 °C and they were exposed to tested agents for 48 h afterwards, followed by the addition of an equal volume (100 μL) of complete culture medium only in the well containing the controls, or twice the final drug concentrations diluted in complete culture medium in the wells where the compounds are tested. Drug activity was measured by means of a SRB colorimetric assay giving the percent of the survival cells towards the control (untreated cells) absorbance. The culture medium was aspirated before fixation and 50 μL of 10% cold trichloroacetic acid (TCA) were gently added to the wells. Microplates were left for 30 min at 4 °C, washed five times with deionized water and left to dry at room temperature for at least 24 h. Subsequently, 70 μL of 0.4% (*w*/*v*) sulforhodamine B (Sigma, Darmstadt, Germany) in 1% acetic acid solution was added to each well and left at room temperature for 20 min. SRB was removed and the plates were washed five times with 1% acetic acid before air-drying. Bound SRB was solubilized with 200 μL of 10 mM un-buffered Tris-base solution. Absorbance was read in a 96-well plate reader at 540 nm.

Evaluation of genotoxicity by micronucleus assay, cell cycle studies, detection of the loss of the Mitochondrial Membrane Permeabilization (MMP assay), and fluorescence spectral studies were performed as previously reported [37]. However, they are all quoted here in brief: (a) Micronucleus*:* MRC-5 cells were seeded (at a density of 2 × 10^4^ cells/well) in glass cover slips which were afterwards placed in six-well plates, with 3 mL of cell culture medium and incubate for 24 h. MRC-5 cells exposed with **1** and **2** in IC_50_ values for a period of 48 h. After the exposure of **1** and **2**, the cover slips were washed three times with PBS and with a hypotonic solution (75 Mm KCl) for 10 min at room temperature. The hypotonized cells were fixed by at least three changes of 1/3 acetic acid/methanol. The cover slips were also washed with cold methanol containing 1% acetic acid. The cover slips were stained with acridine orange (5 μgr/mL) for 15 min at 37 °C. After, the cover slips were rinsed three times with PBS to remove any excess acridine orange stain. The number of micronucleated cells per 1000 cells was determined. (b) Cell cycle: MCF-7 cells were seeded at a density of 10^5^ cells/well in six-well plates at 37 °C for 24 h. Cells were treated with **1** and **2** at the indicated IC_50_ values for 48 h. The cells were then trypsinized and washed twice with phosphate-buffered saline (PBS) and separated by centrifugation. With the addition of 1 mL of cold 70% ethanol, the cells were incubated overnight at −20 °C. For analysis, the cells were centrifuged and transferred into PBS, incubated with RNase (0.2 mg/mL) and propidium iodide (0.05 mg/mL) for 40 min at 310 K and then analyzed by flow cytometry using a FACS Calibur flow cytometer (Becton Dickinson, San Jose, CA, USA). For each sample, 10,000 events were recorded. The resulting DNA histograms were drawn and quantified using the FlowJo software (version FlowJo X 10.0.7r2, Tree Star, Ashland OR, USA). (c) Detection of the loss of the Mitochondrial Membrane Permeabilization: MCF-7 cells were treated with **1** and **2** at IC_50_ values. After 48 h of incubation period of **1** and **2**, the cell medium was removed and added the Dye Loading Solution. The cells were incubated in 5% CO_2_ at 37 °C for 30 min. Afterwards, 50 μL of Assay Buffer B was added of each well and are incubated for 30 min. The fluorescence intensity is measured at λ_ex_ = 540 and λ_em_ = 590 nm. The MMP assay kit used was purchased from sigma Aldrich “Mitochondria Membrane Potential Kit for Microplate Readers, MAK147”. (d) Fluorescent studies: The fluorescence spectroscopy method using ethidium bromide (EB) was employed to determine the relative DNA binding properties of complexes **1** and **2** into CT-DNA. The emission data at 610 nm of the spectra of EB (2.3 μΜ) solutions which contain CT-DNA (26 μΜ) in the absence or presence of various concentrations of complexes **1** and **2** (0–250 μΜ) were recorded upon their excitation at 532 nm (Figure 5). The apparent binding constant (*K*_app_) was calculated using the equation:

*K*_EB_[EB] = *K*_app_[drug]

Where [drug] is the concentration of the complex at a 50% reduction of the fluorescence, *K*_EB_ = 10^7^ M^−1^, and [EB] = 2.3 μΜ. The concentration of the drug at a 50% reduction of the fluorescence is calculated from the diagram *I*_o_/*I* vs. the concentration of the complex [Q] (Figure 5), where, *I*_o_ and *I* are the fluorescence intensities of the CT-DNA in the absence and presence of complexes **1** and **2**, respectively.

The fitting of the experimental *I_o_/I_x_* ratio as a function of concentration (*C*) was performed utilizing the linear least-squares fitting algorithm. However, the fitting procedure depends strongly on the weighting of the data points, i.e., on the knowledge of the standard deviation (SD) of every point in the experimental spectrum (<1%) since all spectra represent the average of 5000 individually recorded spectra. As a measure for the goodness of the fit, we used the *R^2^* statistics.

The slope of the *I_o_/I_x_* ratio as a function of *C* was estimated equal to *a_1_* = 2100.9 ± 204.8 and *a_2_* = 3142.4 ± 561.4 for **1** and **2** case, respectively.

The SD of the *K*_app_ is calculated using the standard error propagation variance formula [50]:

$$s_{f}=\sqrt{{\left(\frac{\partial f}{\partial x}\right)}^{2}{\left(s_{x}\right)}^{2}+{\left(\frac{\partial f}{\partial y}\right)}^{2}{\left(s_{y}\right)}^{2}+{\left(\frac{\partial f}{\partial z}\right)}^{2}{\left(s_{z}\right)}^{2}+\cdots }$$

In general, *s_f_*represents the standard deviation of the function *f*, *s_x_* represents the standard deviation of *x*, *s_y_* represents the standard deviation of *y*, and so forth. This formula is based on the linear characteristics of the gradient of *f* and therefore it is a good estimation for the standard deviation of *f*. Using the above methodology, the parameter *K*_app_ is calculated as 48,950.97 ± 4771.84 and 73,217.92 ± 13,080.62 M^−1^ for **1** and **2**, respectively. More details concerning the calculation of *R^2^*, the error propagation issue and other statistical parameters, see e.g., reference [50]

### 3.5. Viscosity Measurements

The kinematic viscosity of **1** and **2** was measured with the use of an Ubbelohde-type glass capillary viscosity-meter. The value of kinematic viscosity was measured as an average from triplicate measurements of viscosity with an accuracy of 2% at constant temperature. Temperature was set at 298 K by means of an ultra-thermostat.

### 3.6. Computational Studies—Molecular Docking

Molecular docking studies were carried out with AutodockVina [51] along with the graphical interface AutodockTools (ADT 1.5.6) [52] using the Lamarckian genetic algorithm (LGA). The DNA structure (PDB file 1DSC) was considered as a rigid target while no torsional restraints were applied to the complexes. Docking was performed to the optimized organotin derivatives [R_3_Sn(CH_3_COO)], (R = *n*-Bu (**1**), Ph– (**2**)) at the B3LYP/3-21G*/LANL2DZ(Sn) level using the Gaussian03Wsoftware package [53]. Prior to docking, water molecules and the co-crystallized ligand were removed. The search space was set as a grid box of 40 × 30 × 40 Å and spacing of 1 Å centered at the CG rich area. Default parameters were used as described in the Vina manual except from “exhaustiveness” which was set to 24.

## 4. Conclusions

The discovery of the antitumor activity of organotin compounds goes back to the 1980s when Gielen first reports on the subject [8,9,11,12]. Later on, the antitumor properties of orgnaotin compounds were reviewed by Saxena and Hubert [10] and Hadjiliadis et al. [13]. Continuing our studies on the antitumor activity of organotin compounds, two known compounds of formula {[R_3_Sn(CH_3_COO)]*_n_*} where R = *n*-Bu– (**1**) and R = Ph (**2**) with trigonal bipyramidal geometry around each metal center, were used to investigate their antitumor activity against MCF-7 (positive to ERs) and MDA-MB-231 (negative to ERs). The mechanism of their action against human breast tumor cells was also investigated. Compounds **1** and **2** inhibit strongly both cell lines, while their IC_50_ values are lei in nanomolar range (Table 1). The estrogen receptors play no significant role in their action. It is also concluded that (i) the tri-organotin derivatives exhibit stronger activity than the corresponding di-organotin ones (Table 1); (ii) the organotin derivatives of carboxylic acids are generally more active than those of other type of ligands (Table 1); (iii) organotin compounds are also highly toxic against normal MRC-5 cells (Table 1); (iv) the selectivity index, however, shows that **1** and **2** are more potent therapeutics (TPI values of 0.88(**1**) and 0.52(**2**)) than cisplatin (TPI = 0.20) (Table 1) and (v) among all organotins tested from our group the strongest activity against MCF-7 cells has been obtained from the triphenyltin derivative of 2-mercapto-nicotinic acid {[Ph_3_Sn]_2_(mna)·[(CH_3_)_2_CO]} (IC_50_ = 30 nM) and the tri-*n*-butyl tin compound of thiobarbituric acid (*n*-Bu)_3_Sn(o-HTBA)(H_2_O) (IC_50_ = 68 nM and TPI = 1.6) (Table 1). The mitochondrial membrane permeability loss is not supported by MMP assay. Since **1** and **2** are acting through apoptosis on tumor cells (cell cycle studies) this should therefore be activated through a different pathway than that of the mitochondrion, such as the extrinsic apoptotic pathway or direct interaction with DNA. The relative viscosity measurements and fluorescence spectroscopic studies of DNA solutions suggest an intercalation mode of interaction between **1** and **2** and CT-DNA. Docking studies verify the intercalative mode, while the bulk and agile *n*-butyl groups significantly increase the steric hindrance prohibiting the intercalation, resulting in higher antitumor activity of the phenyl derivative instead of the corresponding *n*-Bu– (Table 1).

## Figures and Tables

**Table 1:** Bioactivity data recorded for **1** and **2** in comparison with those of other reported organotin compounds.

Compound	IC_50_ (μΜ)				
	MCF-7	MDA-MB-231	MRC-5	TPI *	Ref.
**1**	0.25 ± 0.02	0.20 ± 0.01	0.22 ± 0.01	0.88	[present]
**2**	0.21 ± 0.01	0.12 ± 0.01	0.11 ± 0.01	0.52	[present]
{[Ph_3_Sn]_2_(mna)·[(CH_3_)_2_CO]}	0.030		>0.200		[18]
[Me_2_Sn(Sal)_2_]	0.142 ± 0.043		0.0975 ± 0.00015	0.69	[19]
[(*n*-Bu)_2_Sn(Sal)_2_]	0.108 ± 0.0026		0.1041 ± 0.0002	0.96	[19]
[(*n*-Bu)_3_Sn(Sal)]	0.724 ± 0.0054		0.0981 ± 0.0001	0.14	[19]
[Ph_3_Sn(Sal)]	0.121 ± 0.0037		0.0945 ± 0.000.2	0.78	[19]
[(*n*-Bu)_3_Sn(pHbza)]	0.325 ± 0.0023		0.0784 ± 0.0002	0.24	[19]
{[Ph_3_Sn(*o*-HTBA)]}*_n_*	0.103	0.203	0.130	1.26	[17]
(*n*-Bu)_3_Sn(*o*-HTBA)(H_2_O)	0.068	0.106	0.108	1.59	[17]
[(tert-Bu–)_2_(HO–Ph)]_2_SnCl_2_	3.12 ± 0.38				[14]
[(tert-Bu–)_2_(HO–Ph)]_2_Sn(PMT)_2_	7.86 ± 0.87				[14]
[(tert-Bu–)_2_(HO–Ph)]_2_Sn(MPMT)_2_	0.58 ± 0.1				[14]
{[(tert-Bu–)_2_(HO–Ph)]_2_SnCl(PYT)}	>30				[14]
[(tert-Bu–)_2_(HO–Ph)]_2_SnCl(MBZT)}	>30				[14]
Ph_3_SnCl	0.130	0.166	0.141	1.08	[16]
[Ph_3_SnOH]*n*	0.070	0.165	0.090	1.29	[16]
[(Ph_2_Sn)_4_Cl_2_O_2_(OH)_2_]	>10	>10	>10		[16]
Me_2_Sn((tert-Bu–)_2_(HO–Ph–S))_2_	19.20 ± 1.70		19.50 ± 1.40	1.02	[15]
Et_2_Sn(((tert-Bu–)_2_(HO–Ph–S))_2_	6.20 ± 0.80		7.30 ± 0.60	1.18	[15]
(*n*-Bu)_2_Sn–(((tert-Bu–)_2_(HO–Ph–S))_2_	0.40 ± 0.06		0.61 ± 0.07	1.53	[15]
Ph_2_Sn(((tert-Bu–)_2_(HO–Ph–S))_2_	6.20 ± 0.80		12.40 ± 1.40	2.00	[15]
[(tert-Bu–)_2_(HO–Ph)]_2_Sn(((tert-Bu–)_2_(HO–Ph–S))_2_	>30	>30	>30		[15]
Me_3_Sn((tert-Bu–)_2_(HO–Ph–S))	4.90 ± 0.50		3.36 ± 0.13	0.69	[15]
Ph_3_Sn(((tert-Bu–)_2_(HO–Ph–S))	0.25 ± 0.03		0.22 ± 0.01	0.88	[15]
Cisplatin	5.5 ± 0.4	26.7 ± 1.1	1.1 ± 0.2	0.20	[37]

* TPI = IC_50_(MRC-5)/IC_50_(MCF-7), mna = 2-mercapto-nicotinic acid, salH = salicylic acid, pHbzaH = *p*-Hydroxyl-benzoic acid, H_2_TBA = 2-thiobarbituric acid, PMTH = 2-mercapto-pyrimidine, MPMTH = 2-mercapto-4-methyl-pyrimidine, PYTH = 2-mercapto-pyridine, MBZTH = 2-mercapto-benzothiazole.

**Table 2:** Cell cycle studies data of **1** and **2**.

Description	Phases of cell cycle			
	Sub-G1	G_0_/G_1_	S	G_2_/M
Untreated cells	6.1%	46.5%	18.3%	28.9%
Treated cells with DMSO	6.5	42.7	20.6	29.5
**1**	14.4	37.9%	25.1	22.1
**2**	24.1	35.8%.	22.9	17.1

## Supplementary Materials

Supplementary materials can be found at http://www.mdpi.com/1422-0067/19/7/2055/s1.
