# Supplementary material for: Poly Organotin Acetates against DNA with Possible Implementation on Human Breast Cancer
Source: Int J Mol Sci. 2018 Jul 14;19(7):2055. doi: 10.3390/ijms19072055 (PMC6073380; doi:10.3390/ijms19072055)
Supplement: Supplementary file 1 [file ijms-19-02055-s001.pdf]

Supplementary Material Information
Poly Organotin Acetates against DNA with Possible Implementation on Human Breast Cancer

G.K. Latsis[1], C.N. Banti[1,*], N. Kourkoumelis[2,*], C. Papatriantafyllopoulou[3], N. Panagiotou[3], A. Tasiopoulos[3], A. Douvalis[4], A.G. Kalampounias[5], T. Bakas[4], S.K. Hadjikakou[1,*]

[1] Section of Inorganic and Analytical Chemistry, Department of Chemistry, University of Ioannina, 45110 Ioannina, Greece

[2] Medical Physics Laboratory, Medical School, University of Ioannina, Greece

[3] Department of Chemistry, University of Cyprus, 1678 Nicosia, Cyprus

[4] Mössbauer Spectroscopy and Physics of Material Laboratory, Department of Physics, University of Ioannina, Greece

[5] Physical Chemistry Laboratory, Department of Chemistry, University of Ioannina, Greece

* All correspondence should be addressed to: Dr C.N. Banti (Post Doctoral Fellow); e-mail: cbanti@cc.uoi.gr; Dr. N. Kourkoumelis (Assistant Professor); e-mail: nkourkou@uoi.gr;
Professor Dr S.K. Hadjikakou; e-mail: shadjika@uoi.gr; tel. xx30-26510-08374

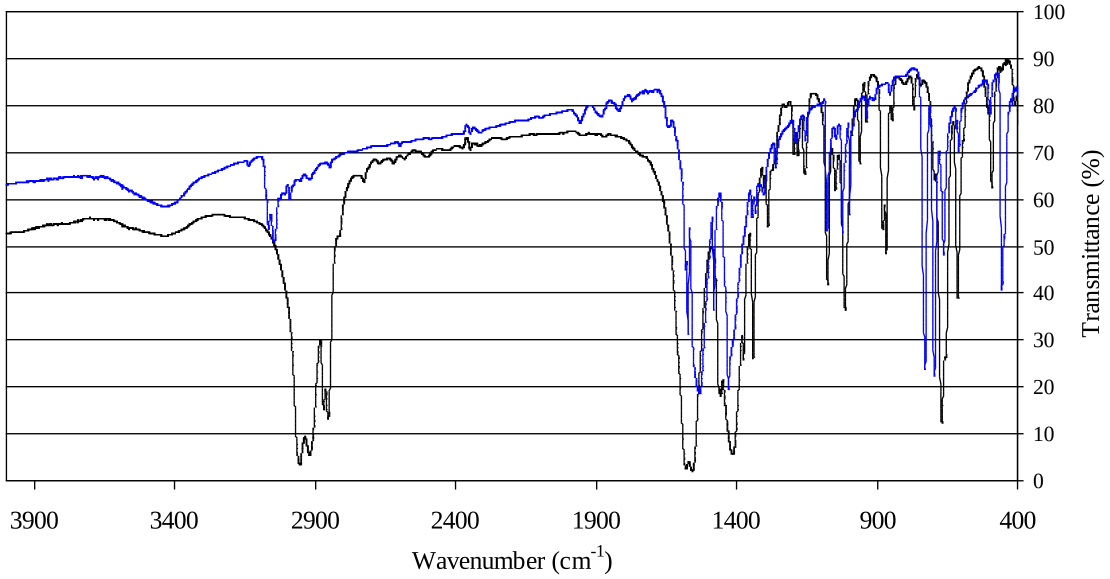

Figure S1. FT-IR spectra of **1** (——) and **2** (——).

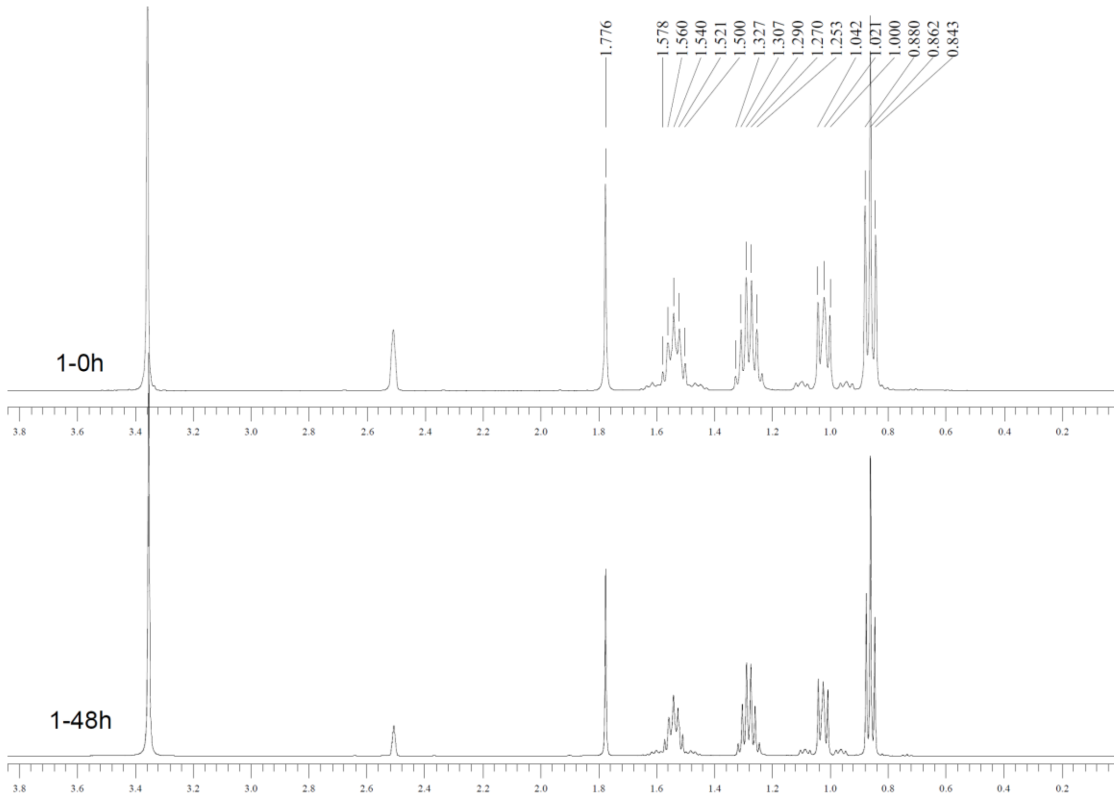

(**A**)

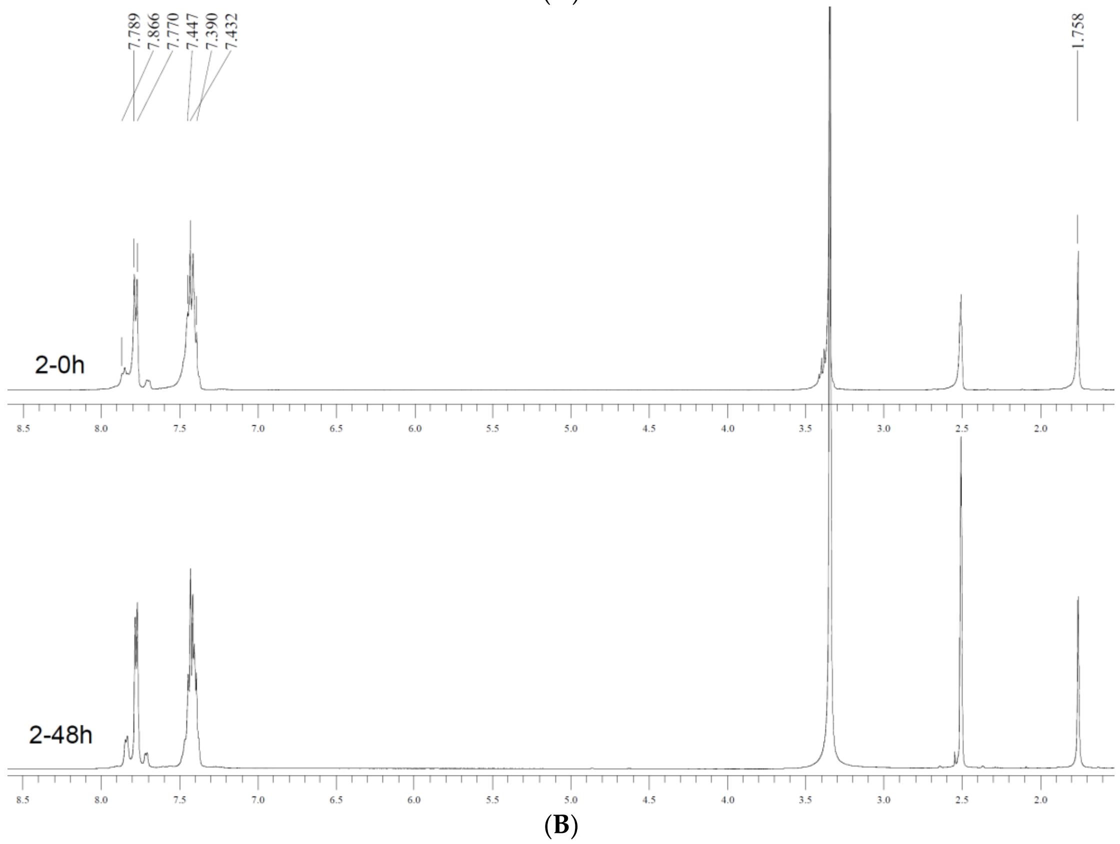

(**B**)

Figure S2. ^{1}H-NMR spectra of **1** (**A**) and **2** (**B**).

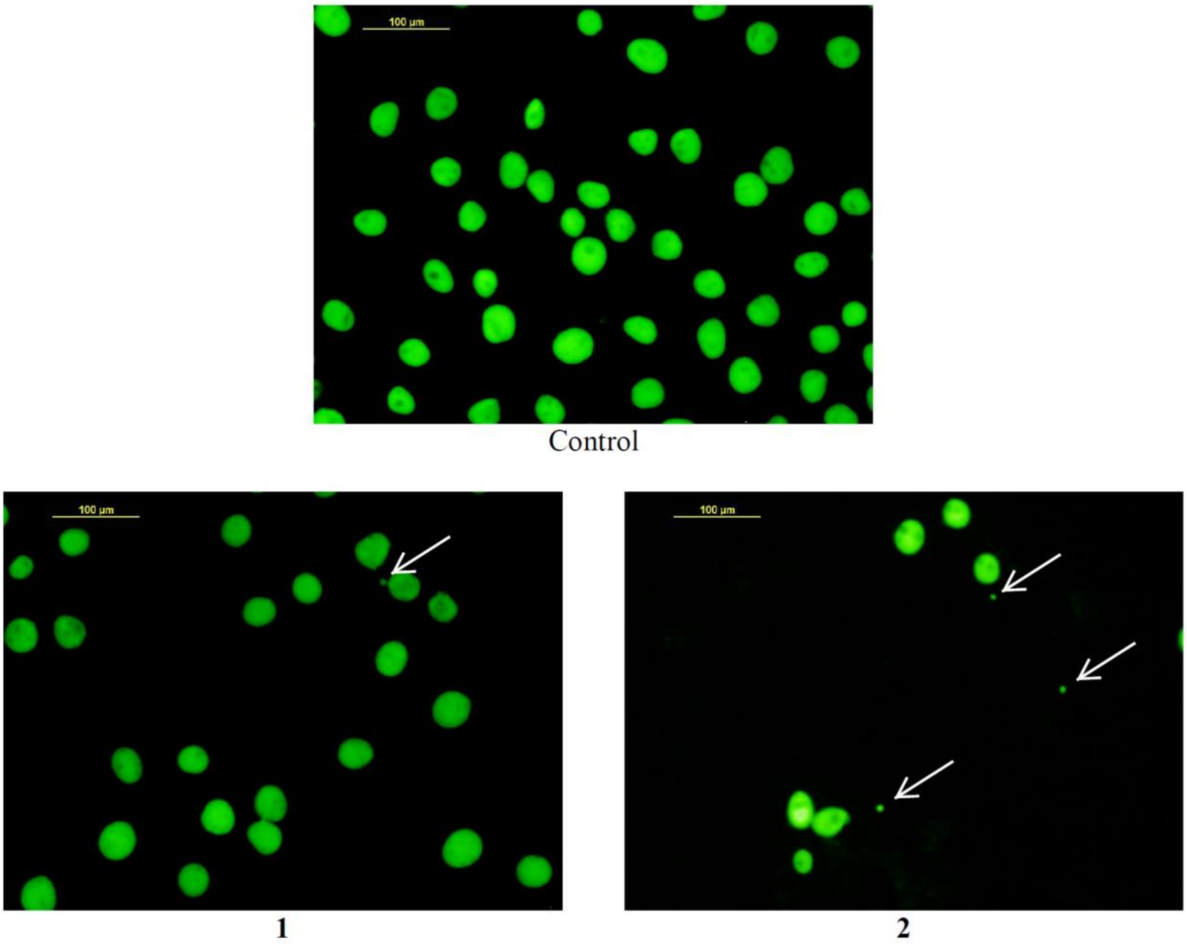

Figure S3. Micronucleus observed in the culture of MRC-5 cells in untreated and treated with **1** and **2** at their IC_{50} value for a period of 48 h, arrow indicates micronucleus in MRC-5 cells.
